# Genetic Polymorphisms of *GSTM1*, *GSTT1*, and *GSTP1* with Prostate Cancer Risk: A Meta-Analysis of 57 Studies

**DOI:** 10.1371/journal.pone.0050587

**Published:** 2012-11-26

**Authors:** Mancheng Gong, Wenjing Dong, Zhirong Shi, Yangyang Xu, Wenjun Ni, Ruihua An

**Affiliations:** 1 Department of Urological Surgery, The First Affiliated Hospital of Harbin Medical University, Harbin, China; 2 Department of Oncology, The First Affiliated Hospital of Harbin Medical University, Harbin, China; 3 Department of Pharmacy, The Second People’s Hospital of Zhuhai, Zhuhai, China; 4 Department of Urological Surgery, The Affiliated Tumor Hospital of Harbin Medical University, Harbin, China; 5 Department of Urological Surgery, The Hei Longjiang Hospital, Harbin, China; IPO, Inst Port Oncology, Portugal

## Abstract

**Background and Objectives:**

The *GSTM1, GSTT1* and *GSTP1* polymorphisms might be involved in inactivation of procarcinogens that contribute to the genesis and progression of cancers. However, studies investigating the association between *GSTM1, GSTT1* or *GSTP1* polymorphisms and prostate cancer (PCa) risk report conflicting results, therefore, we conducted a meta-analysis to re-examine the controversy.

**Methods:**

Published literature from PubMed, Embase, Google Scholar and China National Knowledge Infrastructure (CNKI) were searched (updated to June 2, 2012). According to our inclusion criteria, studies that observed the association between *GSTM1*, *GSTT1* or *GSTP1* polymorphisms and PCa risk were included. The principal outcome measure was the odds ratio (OR) with 95% confidence interval (CI) for the risk of PCa associated with *GSTM1*, *GSTT1* and *GSTP1* polymorphisms.

**Results:**

Fifty-seven studies involving 11313 cases and 12934 controls were recruited. The overall OR, which was 1.2854 (95% CI = 1.1405–1.4487), revealed a significant risk of PCa and *GSTM1* null genotype, and the similar results were observed when stratified by ethnicity and control source. Further, the more important is that the present study first reported the high risks of PCa for people who with dual null genotype of *GSTM1* and *GSTT1* (OR = 1.4353, 95% CI = 1.0345–1.9913), or who with *GSTT1* null genotype and *GSTP1* A131G polymorphism (OR = 1.7335, 95% CI = 1.1067–2.7152). But no association was determined between *GSTT1* null genotype (OR = 1.102, 95% CI = 0.9596–1.2655) or *GSTP1* A131G polymorphism (OR = 1.0845, 95% CI = 0.96–1.2251) and the PCa risk.

**Conclusions:**

Our meta-analysis suggested that the people with *GSTM1* null genotype, with dual null genotype of *GSTM1* and *GSTT1*, or with *GSTT1* null genotype and *GSTP1* A131G polymorphism are associated with high risks of PCa, but no association was found between *GSTT1* null genotype or *GSTP1* A131G polymorphism and the risk of PCa. Further rigorous analytical studies are highly expected to confirm our conclusions and assess gene-environment interactions with PCa risk.

## Introduction

Prostate cancer (PCa) has become a major public health problem concern worldwide for its high morbidity and mortality levels. It is the second leading cause of cancer related to death in Europe, North America, Latin America, and some parts of Africa in men. It has been reported that PCa have a prominent variation in incidence among different ethnic groups and geographic regions. For instance, North Americans have the highest incidence, especially the African-Americans in USA, and the lowest is among Asian men [1]–[3]. However, the etiology and ethnic disparities of PCa are largely unknown. Clinical and epidemiologic data suggest that the development of PCa is a multiphase process. So far, a series environmental and lifestyle factors, including pollutants, smoking habit and diet, as well as geographical and racial factors have been pointed out as possible contributors to the risk of PCa [4]. In addition, the various risk, incidence, and mortality rates among worldwide of PCa suggest that genetic factors also play an important role in PCa initiation and progression, such as individual differences in the susceptibility to cancers, age and family history [5]. Therefore, the occurrence and development of PCa most likely involve a complex interplay between genetic and environmental factors. More specifically, variations in carcinogen metabolism genes may play a critical role in PCa development due to their activation or detoxification functions.

**Table 1:** Characteristics of eligible studies in the meta-analysis of *GSTM1, GSTT1* and *GSTP1* polymorphisms with PCa.

			*GSTM1*			*GSTT1*			*GSTP1*			
First author	Year	Source	Cases^a^	Controls^a^	BPH^a^	Cases^a^	Controls^a^	BPH^a^	Cases^a^	Controls^a^	BPH^a^	*P* value for HWE
**Caucasians**												
Harries LW	1997	HB							10/26	79/76		0.440
Rebbeck TR	1999	PB	110/126	110/121		46/186	72/159					
Wadelius M	1999	PB							75/68	71/49		0.321
Autrup JL	1999	PB	91/62	154/134		29/124	44/244		72/81	131/157		0.932
Steinhoff C	2000	HB	45/46	57/70		23/68	17/110		47/44	70/57		0.390
Shepard TF	2000	HB							290/300	365/438		0.893
Gsur A	2001	BPH	75/91		81/85	27/139		33/133	90/57		65/76	0.258
Kote-Jarai Z	2001	PB	153/120	135/135		67/206	66/212		117/156	140/133		0.215
Luscombe CJ	2002	BPH							86/123		66/88	0.883
Beer TM	2002	PB	61/50	73/74		28/83	33/113		51/58	63/83		0.431
Jeronimo C	2002	mixed#							45/60	61/80		0.374
Kidd LC	2003	/	84/116	100/88		24/178	29/160		92/78	95/73		NA
Nam RK	2003	HB	235/248	266/282		90/393	127/421		227/256	286/262		0.052
Acevedo C	2003	BPH	37/65		29/99							
Debes JD	2004	PB							369/545	184/298		0.310
Medeiros R	2004	PB	77/65	91/92		31/114	44/140					
Mao GE	2004	HB							56/66	70/65		0.622
Joseph MA	2004	PB	97/81	142/123		55/122	61/204					
Mittal RD	2004	BPH	55/48		35/82	35/68		13/104				
Antognelli C	2005	BPH							172/212		220/140	0.498
Caceres DD	2005	PB	37/65	30/102		6/94	14/115					
Srivastava DSL	2005	/	70/57	51/93		41/86	29/115		46/81	83/61		0.227
			***GSTM1***			***GSTT1***			***GSTP1***			
**First author**	**Year**	**Source**	**Cases** a	**Controls** a	**BPH** a	**Cases** a	**Controls** a	**BPH** a	**Cases** a	**Controls** a	**BPH** a	***P*** ** value for HWE**
Vijayalakshmi K	2005	HB	18/57	15/85					49/26	43/57		0.069
Agalliu I	2006	PB	311/248	248/274		92/466	88/434		249/309	226/297		0.662
Quinones LA	2006	HB	22/38	36/81								
Silig Y	2006	HB	98/54	52/117		34/118	31/138					
Rybicki BA	2006	HB							157/206	53/87		0.402
Mittal RD	2006	BPH	31/23		38/67	24/30		30/75	17/37		58/47	0.451
Lima MM Jr	2008	BPH	69/56		53/47	42/83		22/78	65/60		55/45	0.057
Sivonová M	2009	PB	69/60	130/98		24/105	45/183		56/79	110/123		<0.001
Steinbrecher A	2010	PB	126/122	270/221		44/204	77/415		125/123	216/276		0.276
Kumar V	2011	HB+BPH	34/23	15/31	21/32	29/28	22/24	32/21				
Thakur H	2011	HB+BPH	87/63	62/110	82/68	39/111	22/150	18/132				
Rodrigues IS	2011	PB	71/83	86/68		42/112	40/114					
Qadri Q	2011	PB+BPH							26/24	59/21	22/23	0.083
Hemelrijck MV	2012	PB	105/98	188/172		35/168	64/296		100/103	158/202		0.263
**Asians**												
Murata M	2001	BPH	57/58		115/85	47/68		104/96				
Nakazato H	2003	HB	38/43	53/52		40/41	44/61		57/24	76/29		0.101
Aktas D	2004	BPH	19/81		14/93							
Guan TY	2005	PB	48/35	48/67								
Komiya Y	2005	PB	93/93	157/131		74/112	139/149		143/44	212/79		0.148
Wang YL	2005	PB	44/37	40/50		43/38	48/42					
Lai MT	2005	HB	57/39	55/66								
			***GSTM1***			***GSTT1***			***GSTP1***			
**First author**	**Year**	**Source**	**Cases** a	**Controls** a	**BPH** a	**Cases** a	**Controls** a	**BPH** a	**Cases** a	**Controls** a	**BPH** a	***P*** ** value for HWE**
Yang J	2006	HB	99/64	112/90		89/74	95/107					
Wang YL	2008	PB							41/40	58/32		0.786
Li M	2008	HB	121/87	96/134								
Ansari BS	2009	PB	34/26	25/35		13/47	9/51					
Xu XX	2010	PB							68/35	70/33		0.921
Kwon DD	2011	PB	90/76	125/202		85/81	163/164		117/49	209/118		0.300
Ashtiani ZO	2011	PB+BPH	50/60	10/90	47/52	38/72	47/53	37/62				
Safarinejad MR	2011	PB	72/96	94/242		58/110	70/266		54/114	174/162		<0.001
**Africans**												
Mallick S	2007	HB	26/108	36/98		30/104	49/85					
Lavander NA	2009	PB	47/141	137/441		36/153	102/482		55/135	186/386		0.540
Souiden Y	2010	PB	58/52	68/54		30/80	18/104					
**African-Americans**												
Agalliu I	2006	PB	9/22	7/8		7/24	4/11		11/20	1/14		0.019
Rybicki BA	2006	HB							82/192	29/104		0.120
**Mixed**												
Catsburg C	2012	PB	606/774	321/417		242/1158	153/583		569/843	300/449		0.373

a Null/present.

# Used both healthy people and BPH patients as controls.

*GSTM1*, glutathione S-transferase M1; *GSTT1*, glutathione S-transferase T1; *GSTP1*, glutathione S-transferase P1.

PB, population-based controls; HB, hospital-based controls; BPH, benign prostate hyperplasia.

Glutathione S-transferases (GSTs) constitute a superfamily of ubiquitous, multifunctional phase II metabolic enzymes. These enzymes play a crucial function in the detoxification of both endogenous and exogenous carcinogens [6], but also participate in the activation and inactivation of oxidative metabolites of carcinogenic compounds so that to protect DNA from oxidative damage [7]. Hence, it has been speculated that GSTs were probably involved in the development of cancers [8]. As the enzymes are widely distributed in nature and found in essentially all eukaryotic species, individual genetic differences may influence the activity level of GSTs and susceptibility to cancer. To date, the GSTs have been assigned to eight distinct classes: α(*GSTA*),μ(*GSTM*),θ(*GSTT*),π(*GSTP*),σ(*GSTS*),κ(*GSTK*),ο(*GSTO*),τ(*GSTZ*), while several of them are polymorphic that contain one or more homodimer or heterodimer forms [9], [10]. Polymorphisms in these genes, possibly by altering their expression and functional activities, may affect their effect on carcinogen activation/detoxification and DNA repair.

In recent years, *GSTM1*, *GSTT1* and *GSTP1* have been studied most. The *GSTM1*, *GSTT1* and *GSTP1* gene were located on chromosome 1p13.3, 22q11.23, 11q13 respectively [11], [12]. Both *GSTM1* and *GSTT1* gene exhibit an inherited homozygous deletion polymorphism (null genotype), which has been associated with the loss of enzyme activity and increased vulnerability to cytogenetic damage [13]. As a result of decreased efficiency in protection against carcinogens, the individuals with homozygous deletion polymorphism are considered to be at an increased risk for malignancies [10], [14]. Whereas for *GSTP1* polymorphism, a single nucleotide polymorphism in exon 5 (Ile105Val, rs1695) received most attention. The A-to-G transition results in an amino acid change from isoleucine to valine so that leading to significantly lower conjugating activity among individuals who carry one or more copies of the G allele (Ile/Val or Val/Val) compared with those who have the A/A (Ile/Ile) genotype [15]–[17]. Recently, many studies focused on the association between PCa risk and *GSTM1*, *GSTT1* or *GSTP1* polymorphisms, but inconsistent results have been reported. In 2009, Zengnan Mo et al. conducted a meta-analysis [18] suggested that *GSTM1* null genotype conferred an increasing risk of PCa on a wide population basis, but no relationship was found between *GSTT1* and *GSTP1* polymorphisms and the PCa risk. During recent three years, many new researches were performed to study the association between PCa risk and *GSTM1*, *GSTT1* or *GSTP1* polymorphisms, so an updated meta-analysis is needed.

**Figure 1:**
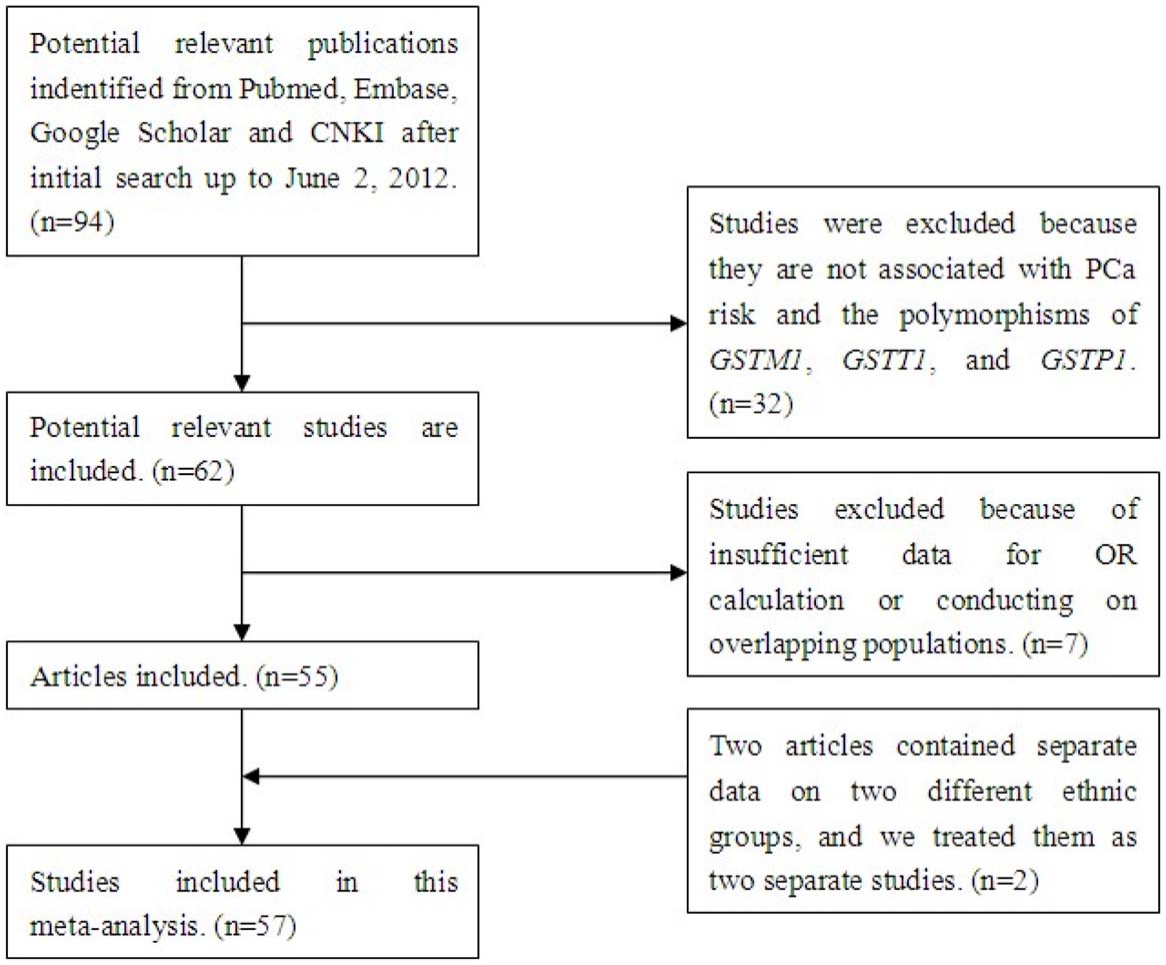
Flow chart of study selection.

**Figure 2:**
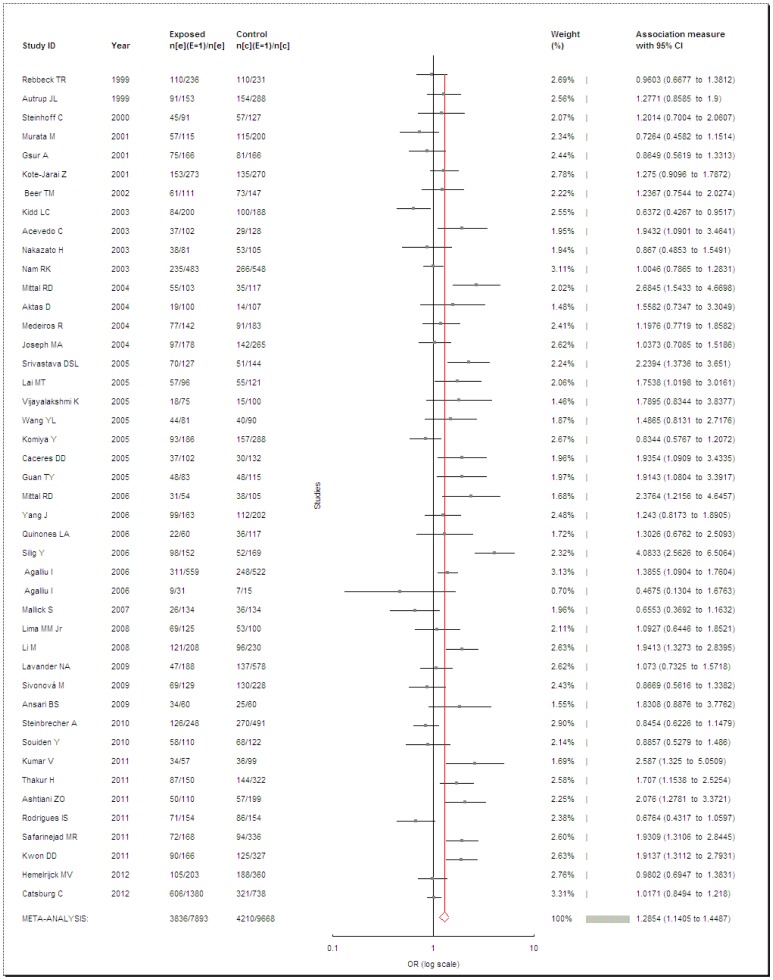
Meta-analysis of *GSTM1* null genotype and PCa risk.

## Materials and Methods

### Search Strategy and Selection Criteria

According to the Preferred Reporting Items for Systematic Reviews and Meta-Analyses (PRISMA) (Checklist S1), we identified all publications (updated to June 2, 2012) by conducting computer-based searches of PubMed, Embase, Google Scholar and China National Knowledge Infrastructure (CNKI). The combination of key words were as follows: ‘glutathione S-transferase M1’ or ‘*GSTM1*’, ‘glutathione S-transferase T1’ or ‘*GSTT1*’, ‘glutathione S-transferase P1’ or ‘*GSTP1*’, ‘prostate’ or ‘urothelial’, ‘cancer’ or ‘carcinoma’ or ‘neoplasm’, ‘polymorphism’ or ‘polymorphisms’. To minimize potential publication bias, no restrictions were placed on language, time period, sample size, type of study and population. All eligible articles were retrieved and their references were checked for other relevant studies. The inclusion criteria were: (1) studies which evaluated associations between *GSTM1, GSTT1, GSTP1* polymorphisms and PCa risk; (2) control population did not contain malignant tumor patients. The exclusion reasons of studies were: (1) insufficient original data for the calculation of odds ratios (ORs) with corresponding 95% confidence intervals (95%CIs); (2) when multiple reports were available for the same study population, we included only the most recent or the largest report. Two investigators independently reviewed the titles, abstracts to determine if an individual study was eligible for the inclusion and exclusion criteria and all disagreements were resolved during a consensus meeting among all reviewers.

### Data Extraction

Table 1 summarized the following information which was extracted from all eligible studies: the name of the first author, year of publication, ethnicity, source of controls, number of cases and controls and *P*-value for Hardy Weinberg Equilibrium (HWE). To ensure the accuracy of extracted information, two independent researchers (Gong and Dong) extracted raw data according to the inclusion criteria. The conflicting evaluations were settled by a discussion among all investigators. Ethnic groups were mainly defined as Caucasian, Asian, African and African-American. Study designs were stratified into three groups: population-based studies, hospital-based studies and benign prostatic hyperplasia (BPH) based studies.

**Figure 3:**
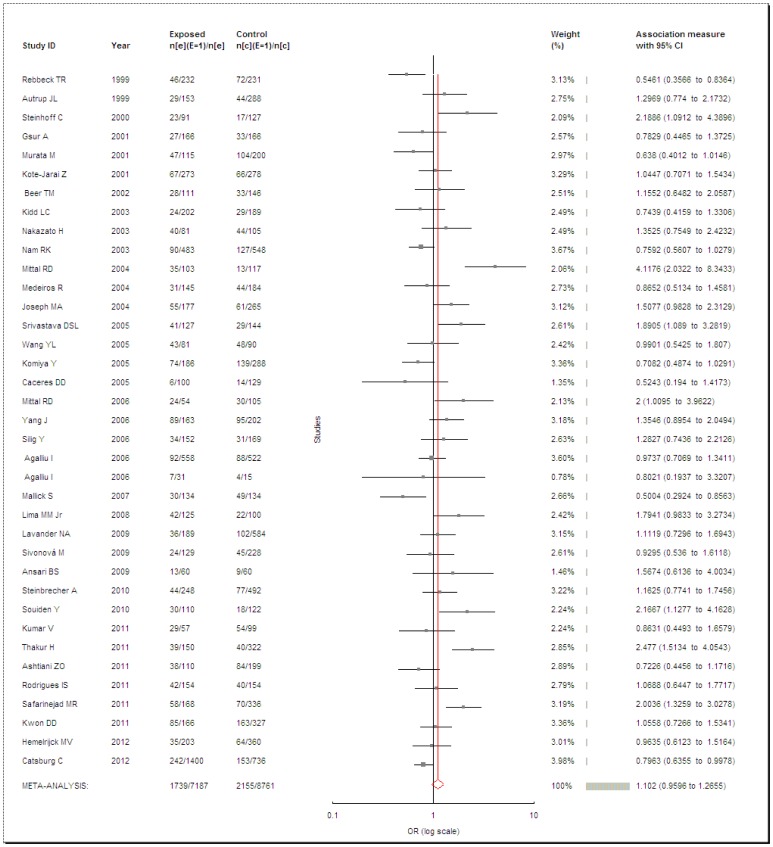
Meta-analysis of *GSTT1* null genotype and PCa risk.

**Figure 4:**
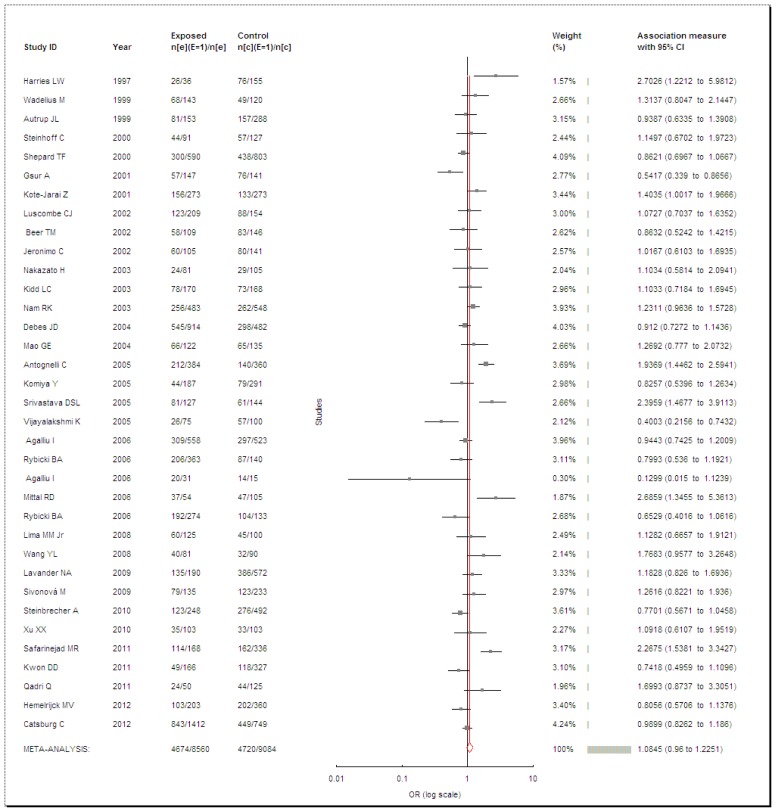
Meta-analysis of *GSTP1* A131G polymorphism and PCa risk.

**Table 2:** Characteristics of eligible studies in the meta-analysis for the combination of *GSTM1, GSTT1* and *GSTP1* polymorphisms with PCa.

			GSTM1+GSTT1				GSTM1+GSTP1		GSTT1+GSTP1		GSTM1+GSTT1+GSTP1	
First author	Year	Source	Both null^a^	Total^a^	Both nulla,b	Totala,b	Both null &AG+GG^a^	Total^a^	Both null &AG+GG^a^	Total^a^	Both null &AG+GG^a^	Total^a^
**Caucasians**												
Rebbeck TR	1999	PB	22/31	468/462								
Autrup JL	1999	PB	19/24	153/288			46/92	153/288	22/24	153/288		
Steinhoff C	2000	HB	8/4	91/127			20/25	91/127	10/5	91/127	1/1	91/127
Kote-Jarai Z	2001	PB									21/16	269/263
Caceres DD	2005	PB	3/5	99/129								
Srivastava DSL	2005	/	23/12	127/144			41/25	127/144	25/14	127/144	14/7	127/144
Vijayalakshmi K	2005	HB					9/11	75/100				
Agalliu I	2006	PB	48/42	558/521			166/145	558/522	48/49	557/522		
Lima MM Jr	2008	BPH			21/9	125/97						
Kumar V	2011	HB+BPH	16/8	57/46	16/12	57/53						
Thakur H	2011	HB+BPH	23/12	150/172	23/10	150/155						
**Asians**												
Nakazato H	2003	HB									5/14	81/105
Safarinejad MR	2011	PB	38/42	168/336			49/49	168/336	36/36	168/336	26/11	168/336
**Africans**												
Souiden Y	2010	PB	11/17	122/110								

a Cases/controls.

b Used BPH patients as controls.

### Statistical Analysis

We used crude ORs with corresponding 95% CIs as a measure of the association between *GSTM1, GSTT1* and *GSTP1* polymorphisms and risk of PCa. The significance of the pooled OR was determined by the *Z* test and *P* value (two-tailed) <0.05 was considered significant. In our study, the *I^2^* test was used to assess the heterogeneity between studies (*I^2^*<25% no heterogeneity; *I^2^* = 25–50% moderate heterogeneity; *I^2^*>50% large or extreme heterogeneity) [19]. The heterogeneity was considered statistically significant with *I^2^*>50% or *P*<0.10. When there was no heterogeneity (*I^2^*≤50% or *P*≥0.10), the fixed-effects model (the Mantel-Haenszel method) was used, otherwise, the random-effects model (the DerSimonian and Laird method) was used when the heterogeneity existed (*I^2^*>50% or *P*<0.10) [20], [21]. Subgroup analyses were performed by ethnicity, source of controls and gene-gene combinations. In addition, sensitivity analysis was performed by omitting each study in turn to assess the stability of results. To determine the evidence of publication bias, the funnel plot and Egger’s test were both used. An asymmetric plot suggested possible publication bias. For the interpretation of Egger’s test, statistical significance was defined as *P*<0.05 [22]. All the statistical analyses were performed with MIX statistical software (Version 1.7 for windows).

**Table 3:** Summary of meta-analysis of *GSTM1, GSTT1* and *GSTP1* polymorphisms and PCa risk.

Groups	No. of studies	No. of subjects	OR (95% CI)	Statistical method	*I^2^*%	*P*-value for *Z* test
**GSTM1**	44	17561	1.2854(1.1405–1.4487)	Random	69.69	<0.0001
Caucasians	26	10134	1.3028(1.1093–1.5301)	Random	72.76	<0.0001
Asians	13	3997	1.4513(1.1682–1.803)	Random	61.46	0.0008
Africans	3	1266	0.9108(0.6943–1.1949)	Fixed	0	0.371
hospital-based studies	12	3821	1.5431(1.1417–2.0856)	Random	78.24	0.0048
population-based studies	23	11091	1.2192(1.0488–1.4172)	Random	68.48	0.0099
BPH-based studies	10	2307	1.3522(1.0067–1.8163)	Random	64.6	0.045
**GSTT1**	37	15948	1.102(0.9596–1.2655)	Random	65.96	0.1119
Caucasians	23	9556	1.1626(0.9712–1.3917)	Random	65.48	0.1006
Asians	9	2937	1.0533(0.8015–1.3842)	Random	65.68	0.7096
Africans	3	1273	1.0465(0.4937–2.2181)	Random	83.85	0.9057
hospital-based studies	8	2814	1.1988(0.8387–1.7135)	Random	73.55	0.3199
population-based studies	22	10919	1.0152(0.8789–1.1727)	Random	51.39	0.8376
BPH-based studies	8	1870	1.3345(0.8308–2.1436)	Random	79.51	0.2327
**GSTP1**	35	17644	1.0845(0.96–1.2251)	Random	69.27	0.1926
**GSTP1** *	32	16726	1.0572(0.9391–1.1902)	Random	65.87	0.3574
Caucasians	25	12230	1.0944(0.9483–1.2629)	Random	70.19	0.2173
Asians	6	2038	1.1924(0.7953–1.7879)	Random	75.57	0.3945
hospital-based studies	9	4361	0.9667(0.7548–1.238)	Random	66.95	0.7883
population-based studies	18	10604	1.0675(0.9221–1.2359)	Random	62.58	0.3817
BPH-based studies	6	1874	1.2012(0.7568–1.9065)	Random	81.31	0.4367
**GSTM1+GSTT1** a	11	4550	1.4353(1.0345–1.9913)	Random	55.91	0.0306
**GSTT1+GSTP1** b	5	2493	1.7335(1.1067–2.7152)	Random	62.42	0.0163
**GSTM1+GSTP1** c	6	2689	1.3867(0.9763–1.9697)	Random	67.33	0.0679
**Three polymorphisms** d	5	1711	1.6903(0.6823–4.1874)	Random	76.3	0.2568

OR, odds ratio; CI, confidence interval.

* *GSTP1* the total result of after excluding three researches deviated from Hardy-Weinberg equilibrium (HWE).

a *GSTM1* (−/−) and *GSTT1* (−/−) vs. *GSTM1* (+/−) and *GSTT1* (−/−) with *GSTM1* (−/−) and *GSTT1* (+/−).

b *GSTT1* (−/−) and *GSTP1* (AG+GG) vs. *GSTT1* (+/−) and GSTP1 (AA) with *GSTT1* (−/−) and GSTP1 (AG+GG).

c *GSTM1* (−/−) and *GSTP1* (AG+GG) vs. *GSTM1* (+/−) and GSTP1 (AA) with *GSTM1* (−/−) and GSTP1 (AG+GG).

d *GSTM1* (−/−), *GSTT1* (−/−) and *GSTP1* (AG+GG) vs. the other combinations of the *GSTM1*, *GSTT1* and *GSTP1* polymorphisms.

## Results

After searching with our eligibility criteria, initially a total of 94 potentially relevant publications were indentified. When screening the title or abstract, 32 studies were excluded because they are not associated with PCa risk and the polymorphisms of *GSTM1*, *GSTT1*, and *GSTP1*. Therefore, we obtained 62 relevant articles that examined the association between the polymorphisms of *GSTM1, GSTT1* or *GSTP1* and PCa risk. Out of them, three studies were excluded because of the insufficient data for OR calculation. Four researches [23]–[26] were eliminated because they were conducted on overlapping populations with other eligible studies [27]–[30]. Hence, 55 studies [27]–[81] met our inclusion criteria and were selected in this meta-analysis. However, one of the eligible studies [61] provided data of both tissue and blood samples from the overlapping population, and we only considered the data of blood samples. In addition, two articles contained separate data on two different ethnic groups [30], [58], and we treated them as two separate studies. Finally, a total of 57 studies were involved in our meta-analysis (Fig.1). The following information was collected from each study: the name of the first author, date of publication, ethnicity, control source, number of cases and controls (Table 1). Most of the researches contained in this meta-analysis were case-control studies, except two nested case-control studies [67], [79] and one cohort study [81]. Among the studies, 44 discussed the association between the *GSTM1* polymorphism and PCa risk, 37 were about *GSTT1*, and 35 were about *GSTP1*. In all eligible studies, there were 26 studies on *GSTM1* genotype of Caucasians, 13 studies of Asians, 3 studies of Africans, 1 study of African-Americans and 1 of mixed populations. Accordingly, 23 studies on *GSTT1* genotype were of Caucasians, 9 studies of Asians, 3 studies of Africans, 1 study of African-Americans and 1 of mixed populations. About *GSTP1* genotype, there were 25 studies of Caucasians, 6 studies of Asians, 2 studies of African-Americans and 1 of mixed populations. According to the control source, 26 were population-based researches, 15 were hospital-based researches, 9 studies were used BPH patients as controls, two were used both healthy people and BPH patients as controls, while the other two studies used hospital-based and BPH patients as controls. In addition, there was one study mixed the healthy people and BPH patients as controls, and the other two were not clarified.

### *GSTM1*

Data from 44 case-control studies comprising 7,893 PCa cases and 9,668 controls were pooled together for analysis of the *GSTM1* polymorphism. The overall data showed that the individuals who carried the *GSTM1* null genotype had a significantly increased PCa risk compared with those who carried the *GSTM1* present genotype in all subjects (OR = 1.2854, 95% CI = 1.1405–1.4487, *P*<0.0001, *I^2^* = 69.69%, Fig. 2). Because the heterogeneity among studies was significant, the random-effects model was conducted. When stratified by ethnicity, the same dramatic risks were found in Caucasians (OR = 1.3028, 95% CI = 1.1093–1.5301, *P* = 0.0013, *I^2^* = 72.76%) and Asians (OR = 1.4513, 95% CI = 1.1682–1.803, *P* = 0.0008, *I^2^* = 61.46%). But it seems that there was no association between PCa risk and the *GSTM1* null genotype in Africans (OR = 0.9108, 95% CI = 0.6943–1.1949, *P* = 0.371, *I^2^* = 0%). When considered the source of the control groups, two studies [43], [55] were excluded for unclear source of controls. Also, high risks were found between PCa and *GSTM1* null genotype in population-based (OR = 1.2192, 95% CI = 1.0488–1.4172, *P* = 0.0099, *I^2^* = 68.48%), hospital-based (OR = 1.5431, 95% CI = 1.1417–2.0856, *P* = 0.0048, *I^2^* = 78.24%) or in BPH-based controls (OR = 1.3522, 95% CI = 1.0067–1.8163, *P* = 0.045, *I^2^* = 64.6%).

### *GSTT1*

Totally, 37 studies met the inclusion criteria and were selected in the meta-analysis with 7,187 cases and 8,761 controls for analysis of the PCa risk and *GSTT1* null genotype. Overall, no enhanced risk was found between the null genotype of *GSTT1* polymorphism and PCa (OR = 1.102, 95% CI = 0.9596–1.2655, *P* = 0.1119, *I^2^* = 65.96%, Fig. 3). As the dramatic heterogeneity, the random-effects model was used. In the subgroup analysis by ethnicity, no associations were observed in Caucasians (OR = 1.1626, 95% CI = 0.9712–1.3917, *P* = 0.1006, *I^2^* = 65.48%), Asians (OR = 1.0533, 95% CI = 0.8015–1.3842, *P* = 0.7096, *I^2^* = 65.68%) or Africans (OR = 1.0465, 95% CI = 0.4937–2.2181, *P* = 0.9057, *I^2^* = 83.85%). In addition, we conducted the subgroup analysis by source of controls with omitting two researches [43], [55] for not clarifying the source of controls. We did not found increased PCa risks with *GSTT1* null genotype in population-based (OR = 1.0152, 95% CI = 0.8789–1.1727, *P* = 0.8376, *I^2^* = 51.39%), in hospital-based (OR = 1.1988, 95% CI = 0.8387–1.7135, *P* = 0.3199, *I^2^* = 73.55%) or in BPH-based controls (OR = 1.3345, 95% CI = 0.8308–2.1436, *P* = 0.2327, *I^2^* = 79.51%).

### *GSTP1*

We obtained 35 articles after searching and data extraction based on our eligibility criteria. In total, 8,560 cases and 9,084 controls were pooled for the association between PCa risk and *GSTP1* A131G polymorphism. However, the result showed no significant risk between PCa and the *GSTP1* A131G polymorphism (OR = 1.0845, 95% CI = 0.96–1.2251, *P* = 0.1926, *I^2^* = 69.27%, Fig. 4). As the heterogeneity was observed, the random-effects model was used. Among the 35 studies, there were three researches deviated from HWE [58], [70], [73], so we excluded them and then obtained another result. Nevertheless, this result (OR = 1.0572, 95% CI = 0.9391–1.1902, *P* = 0.3574, *I^2^* = 65.87%) was similar with the previous one. We also performed subgroup analysis stratified by ethnicity and control source. By ethnicity, we did not acquire remarkable enhanced risks of PCa with *GSTP1* A131G polymorphism either in Caucasians (OR = 1.0944, 95% CI = 0.9483–1.2629, *P* = 0.2173, *I^2^* = 70.19%) or in Asians (OR = 1.1924, 95% CI = 0.7953–1.7879, *P* = 0.3945, *I^2^* = 75.57%). By control source, two studies [43], [55] were eliminated as not mentioned the source of controls. The available data revealed a result that there were no enhanced PCa risks for population-based (OR = 1.0675, 95% CI = 0.9221–1.2359, *P* = 0.3817, *I^2^* = 62.58%), hospital-based (OR = 0.9667, 95% CI = 0.7548–1.238, *P* = 0.7883, *I^2^* = 66.95%) or BPH-based (OR = 1.2012, 95% CI = 0.7568–1.9065, *P* = 0.4367, *I^2^* = 81.31%) controls with the *GSTP1* A131G polymorphism.

### Combination of Genotypes

Several studies reported the combination of *GSTM1*, *GSTT1* and *GSTP1* genotypes (Table 2). For the PCa patients contrast with controls, we detected the remarkable increased PCa risks for people who with dual null genotype of *GSTM1* and *GSTT1* (OR = 1.4353, 95% CI = 1.0345–1.9913, *P* = 0.0306, *I^2^* = 55.91%) and people who with *GSTT1* null genotype and *GSTP1* A131G polymorphism (OR = 1.7335, 95% CI = 1.1067–2.7152, *P* = 0.0163, *I^2^* = 62.42%). However, when combined the *GSTM1* null genotype and *GSTP1* A131G polymorphism (OR = 1.3867, 95% CI = 0.9763–1.9697, *P* = 0.0679, *I^2^* = 67.33%), or the three genotypes (OR = 1.6903, 95% CI = 0.6823–4.1874, *P* = 0.2568, *I^2^* = 76.3%), no dramatic PCa risks were obtained.

### Sensitivity Analyses

Sensitivity analyses were performed by sequential omission of individual studies for all subjects and subgroups. The corresponding pooled ORs were not materially altered in all subjects and subgroups of *GSTM1*, *GSTT1* or *GSTP1* genotypes (data not shown). The results of sensitivity analyses indicated the stability of the results of this meta-analysis.

### Publication Bias

Funnel plot and Egger’s test were both performed to access the publication bias in this meta-analysis. The funnel plot shapes of *GSTM1* and *GSTP1* polymorphisms were symmetrical (data not shown) and the *P* values of Egger’s test were 0.0625 and 0.4738 respectively, so the results showed no evidence of publication biases. However, the shape of *GSTT1* genotype revealed a little unsymmetrical (data not shown), therefore the Egger’s test was further applied to provide statistical evidence and the result suggested the publication bias might be existed, and the *P* value was 0.0415. Hence, we conducted the trim-and-fill in order to get further information. The result revealed that the number of imputed studies was zero, and also the corrected OR was 1.102 (95% CI = 0.9596–1.2655) which was the same as the uncorrected one.

## Discussion

PCa is the most commonly diagnosed non-skin malignancy among men and its incidence is expected to increase as the population age elevated [82]. The molecular genetics of PCa is poorly understood. Its heterogeneous nature suggests that predisposition to PCa may involve multiple genes and variable phenotypic expression. The glutathiones S-transferases (GSTs) are the most important parts of phase II superfamily of metabolism enzymes. In humans, there are several GST classes that are encoded by distinct gene families [83]. Among them, *GSTM1*, *GSTT1* and *GSTP1* should be pointed out because the polymorphisms of these genes may influence the enzyme activity, and eventually increase vulnerability to genotoxic damage [14]. Therefore, the association between the polymorphisms of *GSTM1*, *GSTT1* or *GSTP1* and PCa has been intensively investigated.

In this study, association between *GSTM1, GSTT1* or *GSTP1* genetic variants and PCa risk were examined and all the results of the present meta-analysis were summarized in Table 3. Our result suggested that a significant increased risk existed between PCa and *GSTM1* null genotype, whereas no elevated PCa risks were observed with the *GSTT1* null genotype and *GSTP1* polymorphism. It is consistent with the result of former meta-analysis, which was conducted by Zengnan Mo et al. in 2009. However, we included 11313 cases and 12934 controls from 57 studies in the present meta-analysis, which is much more than the previous one including 7,984 cases and 9,143 controls from 39 case-control studies. Hence, a more stringent and comprehensive result has been obtained.

It is known that the allele frequencies of metabolic genes are not equally distributed throughout the human population but follow diverse ethnic patterns, therefore, the subgroups according to ethnicity were performed. Our results indicated that significant PCa risks of people with *GSTM1* null genotype are in all subjects, especially in Caucasians and Asians, but not in Africans. The possible reason of the conflicting results among diverse ethnicities could be that different genetic backgrounds and environment they exposed to may have different effects on the PCa risk. Additionally, as limited sample size may have not enough statistical power to detect a real effect or generate a fluctuated estimation, the small sample size of Africans in this meta-analysis should also be taken into consideration.

Furthermore, we also showed that *GSTM1* null genotype has strikingly increased the risk of PCa susceptibility when stratified by control source. However, we obtained the highest risk of PCa when only considered the hospital-based controls. The possible reason may be that *GSTM1* null genotype could influence the susceptibility to non-cancer diseases, such as COPD [84], alcoholic liver disease [85], and coronary heart disease [86], so its genotype frequency possibly differed between the hospital-based and population-based controls. Besides, we got a higher PCa risk of BPH-based controls than population controls. For this result, the probably reason could be the selection bias. To be specific, the differences of selection criteria or selection chance between population and BPH-based controls may be the main reasons of the selection bias. On the other hand, we did not exclude that the BPH could be affected by the *GSMT1* null genotype [87] was one of the reasons for the result. However, the exactly reason need to be further confirmed.

In addition, we first observed the association between the combination of *GSTM1, GSTT1* or *GSTP1* genotypes and PCa risk and revealed important results. Eleven articles examined the people with dual null genotype of *GSTM1* and *GSTT1*, and our result proved a remarkable increased PCa risk for these people. Moreover, the result also revealed a very strong risk of PCa for people who with *GSTT1* null genotype and *GSTP1* A131G polymorphism from five articles. The present meta-analysis is the earliest one to evaluate the potential interaction of the gene-to-gene and PCa risk. However, we should treat the results with caution for the limited sample size.

For the *GSTT1* null genotype and *GSTP1* A131G polymorphism, we failed to find the association between PCa risk and the polymorphisms, even though we stratified for ethnicity and control source, which is consistent with the previous meta-analysis [18].

However, there are some limitations in this meta-analysis. First of all, even though we performed subgroup analyses stratified by ethnicity and control source, the heterogeneity for *GSTM1* polymorphism among the studies was extreme. It suggested that there were other potential confounding factors in the included studies, such as the genotyping error, selection bias, or population-specific gene-gene or gene-environment interaction, allelic heterogeneity, or chance [88], [89]. Although evidence of heterogeneity exists, it was found through sensitivity analysis that studies contribute to the heterogeneity do not significantly alter the estimate of overall odds ratio. Secondly, only published studies were included, therefore the publication bias may have been occurred. The Egger’s test provided statistical evidence of that. We observed the publication bias when only considered studies about the association between *GSTT1* polymorphism and PCa risk, but did not find it in the studies about the PCa risks with *GSTM1* and *GSTP1* polymorphisms. It is known that positive results usually have a greater probability of being published, and such bias may occur when studies with null or unexpected results. In addition, we also performed the trim-and-fill and the corrected OR was the same as the uncorrected one. Therefore, our result of *GSTT1* null genotype was reliable and stable to some extent. Thirdly, the overall outcomes were based on unadjusted effect estimates. Although the cases and controls were matched on age, sex and residence in all studies, these confounding factors might slightly modify the effective estimates and a more precise evaluation needed to be adjusted by the potentially suspected factors. Finally, as the meta-analysis remains a retrospective research which is subject to the methodological deficiencies of the included studies, we tried to develop a detailed protocol before initiating the study, and then performed an explicit method for study researching, selection, data extraction and data analysis to minimize the likelihood of bias.

### Conclusions

In conclusion, our meta-analysis suggested that *GSTM1* null genotype is associated with a high increased risk of PCa and no significant PCa risks were obtained for *GSTT1* and *GSTP1* polymorphisms. To our knowledge, the present study is the first meta-analysis to date to report the interaction between the combination of *GSTM1, GSTT1* or *GSTP1* genotypes and PCa risk. In the meta-analysis, we proved remarkable elevated PCa risks for people who with dual null genotype of *GSTM1* and *GSTT1,* and also for people who with *GSTT1* null genotype and *GSTP1* A131G polymorphism. Larger and more rigorous analytical studies will be required to confirm our findings and evaluate gene-environment interactions with PCa risk.

## Supporting Information

Checklist S1 (DOC)

## References

[1] Jemal A, Siegel R, Ward E, Murray T, Xu J, et al (2006) Cancer statistics. CA Cancer J Clin 56: 106–130. 16514137 10.3322/canjclin.56.2.106

[2] Greenlee RT, Hill-Harmon MB, Murray T, Thun M (2001) Cancer statistics. CA Cancer J Clin 51: 15–36. 11577478 10.3322/canjclin.51.1.15

[3] Parkin DM, Bray F, Ferlay J, Pisani P (2002) Global cancer statistics. CA Cancer J Clin 55: 74–108. 10.3322/canjclin.55.2.74 15761078

[4] Fleshner N, Zlotta AR (2007) Prostate cancer prevention: past, present, and future. Cancer 110: 1889–1899. 17893870 10.1002/cncr.23009

[5] American Cancer Society: Cancer Facts & Figures 2009. 2009.

[6] Hayes JD, Pulford DJ (1995) The glutathione S-transferase supergene family: regulation of GST and the contribution of the isoenzymes to cancer chemoprotection and drug resistance. Crit Rev Biochem Mol Biol 30: 445–600. 8770536 10.3109/10409239509083491

[7] Ryberg D, Skaug V, Hewer A, Phillips DH, Harries LW, et al (1997) Genotypes of glutathione transferase M1 and P1 and their significance for lung DNA adduct levels and cancer risk. Carcinogenesis 18: 1285–1289. 9230269 10.1093/carcin/18.7.1285

[8] Rebbeck TR (1997) Molecular epidemiology of the human glutathione S-transferase genotypes GSTM1 and GSTT1 in cancer susceptibility. Cancer Epidemiol Biomarkers Prev 6: 733–743. 9298582

[9] Hayes JD, Strange RC (2000) Glutathione S-transferases polymorphisms and their biological consequence. Pharmacology 61: 154–166. 10971201 10.1159/000028396

[10] Hayes JD, Flanagan JU, Jowsey IR (2005) Glutathione transferases. Annu Rev Pharmacol Toxtcol 45: 51–88. 10.1146/annurev.pharmtox.45.120403.095857 15822171

[11] Pearson WR, Vorachek WR, Xu SJ, Berger R, Hart I, et al (1993) Identification of class-mu glutathione transferase genes GSTM1-GSTM5 on human chromosome 1p13. Am J Hum Genet 53: 220–233. 8317488 PMC1682241

[12] Webb G, Vaska V, Coggan M, Board P (1996) Chromosomal localization of the gene for the human theta class glutathione transferase (GSTT1). Genomics 33: 121–123. 8617495 10.1006/geno.1996.0167

[13] Norppa H (2004) Cytogenetic biomarkers and genetic polymorphisms. Toxicol Lett 149: 309–334. 15093278 10.1016/j.toxlet.2003.12.042

[14] McIlwain CC, Townsend DM, Tew KD (2006) Glutathione S-transferase polymorphisms: cancer incidence and therapy. Oncogene 25: 1639–1648. 16550164 10.1038/sj.onc.1209373 PMC6361140

[15] Ali-Osman F, Akande O, Antoun G, Mao JX, Buolamwini J (1997) Molecular cloning, characterization, and expression in Escherichia coli of full-length cDNAs of three human glutathione S-transferase Pi gene variants. Evidence for differential catalytic activity of the encoded proteins. J Biol Chem. 272: 10004–10012. 10.1074/jbc.272.15.10004 9092542

[16] Hu X, Ji X, Srivastava SK, Xia H, Awasthi S, et al (1997) Mechanism of differential catalytic efficiency of two polymorphic forms of human glutathione S-transferase P1-1 in the glutathione conjugation of carcinogenic diol epoxide of chrysene. Arch Biochem Biophys 345: 32–38. 9281308 10.1006/abbi.1997.0269

[17] Sundberg K, Johansson AS, Stenberg G, Widersten M, Seidel A, et al (1998) Differences in the catalytic efficiencies of allelic variants of glutathione transferase P1-1 towards carcinogenic diol epoxides of polycyclic aromatic hydrocarbons. Carcinogenesis 19: 433–436. 9525277 10.1093/carcin/19.3.433

[18] Mo Z, Gao Y, Cao Y, Gao F, Jian L (2009) An updating meta-analysis of the GSTM1, GSTT1, and GSTP1 polymorphisms and prostate cancer: a HuGE review. Prostate 69: 662–688. 19143011 10.1002/pros.20907

[19] Higgins JP, Thompson SG, Deeks JJ, Altman DG (2003) Measuring inconsistency in meta-analyses. BMJ 327: 557–560. 12958120 10.1136/bmj.327.7414.557 PMC192859

[20] Mantel N, Haenszel W (1959) Statistical aspects of the analysis of data from retrospective studies of disease. J Natl Cancer Inst 22: 719–748. 13655060

[21] DerSimonian R, Laird N (1986) Meta-analysis in clinical trials. Control Clini Trials 7: 177–188. 10.1016/0197-2456(86)90046-2 3802833

[22] Egger M, Davey Smith G, Schneider M, Minder C (1997) Bias in meta-analysis detected by a simple, graphical test. Br Med J 315: 629–634. 9310563 10.1136/bmj.315.7109.629 PMC2127453

[23] Murata M, Shiraishi T, Fukutome K, Watanabe M, Nagao M, et al (1998) Cytochrome P4501A1 and glutathione S-transferase M1 genotypes as risk factors for prostate cancer in Japan. Jpn J Clin Oncol 28: 657–660. 9861231 10.1093/jjco/28.11.657

[24] Kelada SN, Kardia SL, Walker AH, Wein AJ, Malkowicz SB, et al (2000) The glutathione S-transferase-mu and -theta genotypes in the etiology of prostate cancer: genotype-environment interactions with smoking. Cancer Epidemiol Biomarkers Prev 9: 1329–1334. 11142418

[25] Guan TY, Li M, Liu SZ, Li Y, Zhou LQ, et al (2004) CYP1A1 and GSTM1 gene polymorphisms and genetic susceptibility to prostate cancer. Chin J Urol 10: 697–700.

[26] Nock NL, Bock C, Neslund-Dudas C, Beebe-Dimmer J, Rundle A, et al (2009) Polymorphisms in glutathione S-transferase genes increase risk of prostate cancer biochemical recurrence differentially by ethnicity and disease severity. Cancer Causes Control 20: 1915–1926. 19568698 10.1007/s10552-009-9385-0 PMC2777237

[27] Murata M, Watanabe M, Yamanaka M, Kubota Y, Ito H, et al (2001) Genetic polymorphisms in cytochrome P450 (CYP) 1A1, CYP1A2, CYP2E1, glutathione S-transferase (GST) M1 and GSTT1 and susceptibility to prostate cancer in the Japanese population. Cancer Lett 165: 171–177. 11275366 10.1016/s0304-3835(01)00398-6

[28] Rebbeck TR, Walker AH, Julie M, White DL, Wein AJ, et al (1999) Glutathione S-Transferase-m (GSTM1) and -u (GSTT1) Genotypes in the Etiology of Prostate Cancer. Cancer Epidemiol Biomarkers Prev 8: 283–287. 10207629

[29] Guan TY, Li M, Na YQ (2005) Polymorphism of metabolic gene and genetic susceptibility to prostate cancer. Chin J Surg 43: 1467–1470. 16318816

[30] Rybicki BA, Neslund-Dudas C, Nock NL, Schultz LR, Eklund L, et al (2006) Prostate cancer risk from occupational exposure to polycyclic aromatic hydrocarbons interacting with the GSTP1 Ile105Val polymorphism. Cancer Detect Prev 30: 412–422. 17067754 10.1016/j.cdp.2006.09.004 PMC1769317

[31] Debes JD, Yokomizo A, McDonnell SK, Hebbring SJ, Christensen GB, et al (2004) Gluthatione S-transferase P1 polymorphism I105V in familial and sporadic prostate cancer. Cancer Genet Cytogenet 155: 82–86. 15527908 10.1016/j.cancergencyto.2004.03.015

[32] Harries LW, Stubbins MJ, Forman D, Howard GC, Wolf CR (1997) Identification of genetic polymorphisms at the glutathione S-transferase Pi locus and association with susceptibility to bladder, testicular and prostate cancer. Carcinogenesis 18: 641–644. 9111193 10.1093/carcin/18.4.641

[33] Wadelius M, Autrup JL, Stubbins MJ, Andersson SO, Johansson JE, et al (1999) Polymorphisms in NAT2, CYP2D6, CYP2C19 and GSTP1 and their association with prostate cancer. Pharmacogenetics 9: 333–340. 10471065 10.1097/00008571-199906000-00008

[34] Autrup JL, Thomassen LH, Olsen JH, Wolf H, Autrup H (1999) Glutathione S-transferases as risk factors in prostate cancer. Eur J Cancer Prev 8: 525–832. 10643942 10.1097/00008469-199912000-00008

[35] Steinhoff C, Franke KH, Golka K, Thier R, Romer HC, et al (2000) Glutathione transferase isozyme genotypes in patients with prostate and bladder carcinoma. Arch Toxicol 74: 521–526. 11131031 10.1007/s002040000161

[36] Shepard TF, Platz EA, Kantoff PW, Nelson WG, Isaacs WB, et al (2000) No association between the I105V polymorphism of the glutathione S-transferase P1 gene (GSTP1) and prostate cancer risk: A prospective study. Cancer Epidemiol Biomarkers Prev 9: 1267–1268. 11097238

[37] Gsur A, Haidinger G, Hinteregger S, Bernhofer G, Schatzl G, et al (2001) Polymorphisms of glutathione S-transferase genes (GSTP1, GSTM1 and GSTT1) and prostate-cancer risk. Int J Cancer 95: 152–155. 11307147 10.1002/1097-0215(20010520)95:3<152::aid-ijc1026>3.0.co;2-s

[38] Kote-Jarai Z, Easton D, Edwards SM, Jefferies S, Durocher F, et al (2001) Relationship between glutathione S-transferase M1, P1 and T1 polymorphisms and early onset prostate cancer. Pharmacogenetics 11: 325–330. 11434510 10.1097/00008571-200106000-00007

[39] Luscombe CJ, French ME, Liu S, Saxby MF, Farrell WE, et al (2002) Glutathione S-transferase GSTP1 genotypes are associated with response to androgen ablation therapy in advanced prostate cancer. Cancer Detect Prev 26: 376–380. 12518868 10.1016/s0361-090x(02)00089-2

[40] Beer TM, Evans AJ, Hough KM, Lowe BA, McWilliams JE, et al (2002) Polymorphisms of GSTP1 and related genes and prostate cancer risk. Prostate Cancer and Prostatic Diseases 5: 22–27. 15195126 10.1038/sj.pcan.4500549

[41] Jeronimo C, Varzim G, Henrique R, Oliveira J, BentoMJ, et al (2002) I105V polymorphism and promoter methylation of the GSTP1 gene in prostate adenocarcinoma. Cancer Epidemiol Biomarkers Prev 11: 445–450. 12010858

[42] Nakazato H, Suzuki K, Matsui H, Koike H, Okugi H, et al (2003) Association of genetic polymorphisms of glutathione S-transferase genes (GSTM1, GSTT1 and GSTP1) with familial prostate cancer risk in a Japanese population. Anticancer Res 23: 289–902. 12926131

[43] Kidd LC, Woodson K, Taylor PR, Albanes D, Virtamo J, et al (2003) Polymorphisms in glutathione S-transferase genes (GST-M1 GST-T1 and GST-P1) and susceptibility to prostate cancer among male smokers of the ATBC cancer prevention study. Eur J Cancer Prev 12: 317–320. 12883385 10.1097/00008469-200308000-00012

[44] Nam RK, Zhang WW, Trachtenberg J, Jewett MA, Emami M, et al (2003) Comprehensive assessment of candidate genes and serological markers for the detection of prostate cancer. Cancer Epidemiol Biomarkers Prev 12: 1429–1437. 14693733

[45] Acevedo C, Opazo JL, Huidobro C, Cabezas J, Iturrieta J, et al (2003) Positive correlation between single or combined genotypes of CYP1A1 and GSTM1 in relation to prostate cancer in Chilean people. Prostate 57: 111–117. 12949934 10.1002/pros.10274

[46] Medeiros R, Vasconcelos A, Costa S, Pinto D, Ferreira P, et al (2004) Metabolic susceptibility genes and prostate cancer risk in a southern European population: The role of glutathione S-transferases GSTM1, GSTM3, and GSTT1 genetic polymorphisms. Prostate 58: 414–420. 14968442 10.1002/pros.10348

[47] Mao GE, Morris G, Lu QY, Cao W, Reuter VE, et al (2004) Glutathione S-transferase P1 Ile105Val polymorphism, cigarette smoking and prostate cancer. Cancer Detection and Prev 28: 368–374. 10.1016/j.cdp.2004.07.003 15542263

[48] Joseph MA, Moysich KB, Freudenheim JL, Shields PG, Bowman ED, et al (2004) Ambrosone cruciferous vegetables, genetic polymorphisms in glutathione S-transferases m1 and t1, and prostate cancer risk. Nutr Cancer 50: 206–213. 15623468 10.1207/s15327914nc5002_11

[49] Mittal RD, Srivastava DSL, Mandhani A, Kumar A, Mittal B (2004) PolymorphismofGSTM1 and GSTT1 genes in prostate cancer: A study from North India. Indian J Cancer 41: 115–119. 15472409

[50] Aktas D, Hascicek M, Sozen S, Ozen H, Tuncbilek E (2004) CYP1A1 and GSTM1 polymorphic genotypes in patients with prostate cancer in a Turkish population. Cancer Genet Cytogenet 154: 81–85. 15381379 10.1016/j.cancergencyto.2004.01.023

[51] Antognelli C, Mearini L, Talesa VN, Giannantoni A, Mearini E (2005) Association of CYP17, GSTP1, and PON1 polymorphisms with the risk of prostate cancer. Prostate 63: 240–251. 15538743 10.1002/pros.20184

[52] Komiya Y, Tsukino H, Nakao H, Kuroda Y, Imai H, et al (2005) Human glutathione S-transferase A1, T1, M1, and P1 polymorphisms and susceptibility to prostate cancer in the Japanese population. J Cancer Res Clin Oncol 131: 238–242. 15616829 10.1007/s00432-004-0634-z PMC12161227

[53] Lai MT, Chen RH, Tsai FJ, Wan L, Chen WC (2005) Glutathione S-transferaseM1 gene but not insulin-like growth factor-2 gene or epidermal growth factor gene is associated with prostate cancer. Urol Oncol 23: 225–229. 16018936 10.1016/j.urolonc.2005.01.018

[54] Caceres DD, Iturrieta J, Acevedo C, Huidobro C, Varela N, et al (2005) Relationship among metabolizing genes, smoking and alcohol used as modifier factors on prostate cancer risk: Exploring some gene-gene and gene-environment interactions. Eur J Epidemiol 20: 79–88. 15756908 10.1007/s10654-004-1632-9

[55] Srivastava DSL, Mandhani A, Mittal B, Mittal RD (2005) Genetic polymorphism of glutathione S-transferase genes (GSTM1,GSTT1 and GSTP1) and susceptibility to prostate cancer in Northern India. 95: 170–173. 10.1111/j.1464-410X.2005.05271.x 15638917

[56] Vijayalakshmi K, Vettriselvi V, Krishnan M, Shroff S, Vishwa-nathan KN, et al (2005) Polymorphisms at GSTM1 and GSTP1 gene loci and risk of prostate cancer in a South Indian population. Asian Pac J Cancer Prev 6: 309–314. 16235991

[57] Yang J, Wu HF, Zhang W, Gu M, Hua LX, et al (2006) Polymorphisms of metabolic enzyme genes, living habits and prostate cancer susceptibility. Front Biosci 11: 2052–2060. 16720291 10.2741/1947

[58] Agalliu I, Langeberg WJ, Lampe JW, Salinas CA, Stanford JL (2006) Glutathione S-transferase M1, T1, and P1 polymorphisms and prostate cancer risk in middle-aged men. Prostate 66: 146–156. 16173036 10.1002/pros.20305

[59] Quiñones LA, Irarrázabal CE, Rojas CR, Orellana CE, Acevedo C, et al (2006) Joint effect among p53, CYP1A1, GSTM1 polymorphism combinations and smoking on prostate cancer risk: An exploratory genotype-environment interaction study. Asian J Androl 8: 349–355. 16625286 10.1111/j.1745-7262.2006.00135.x

[60] Silig Y, Pinarbasi H, Gunes S, Ayan S, Bagci H, et al (2006) Polymorphisms of CYP1A1, GSTM1, GSTT1, and prostate cancer risk in Turkish population. Cancer Invest 24: 41–45. 16466991 10.1080/07357900500449579

[61] Mittal RD, Mishra DK, Mandhani A (2006) Evaluating polymorphic status of glutathione S-transferase genes in blood and tissue samples of prostate cancer patients. Asian Pacific J Cancer Prev 7: 444–446. 17059341

[62] Mallick S, Romana M, Blanchet P, Multigner L (2007) GSTM1 and GSTT1 polymorphisms and the risk of prostate cancer in a Caribbean population of African descent. Urology 69: 1165–1169. 17572208 10.1016/j.urology.2007.02.039

[63] Li M, Guan TY, Li Y, Na YQ (2008) Polymorphisms of GSTM1 and CYP1A1 genes and their genetic susceptibility to prostate cancer in Chinese men. Chin Med J (Engl) 121: 305–308. 18304461

[64] Ashtiani ZO, Hasheminasab SM, Ayati M, Goulian BS, Modarressi MH (2010) Are GSTM1, GSTT1 and CAG repeat length of androgen receptor gene polymorphisms associated with risk of prostate cancer in Iranian patients? Pathol Oncol Res 17: 269–275. 21089003 10.1007/s12253-010-9309-z

[65] Kumar V, Yadav CS, Datta SK, Singh S, Ahmed RS, et al (2011) Association of GSTM1 and GSTT1 polymorphism with lipid peroxidation in benign prostate hyperplasia and prostate cancer: a pilot study. Dis Markers 30: 163–169. 21694442 10.3233/DMA-2011-0774 PMC3825238

[66] Thakur H, Gupta L, Sobti RC, Janmeja AK, Seth A, et al (2011) Association of GSTM1T1 genes with COPD and prostate cancer in north Indian population. Mol Biol Rep 38: 1733–1739. 20842440 10.1007/s11033-010-0287-8

[67] Steinbrecher A, Rohrmann S, Timofeeva M, Risch A, Jansen E, et al (2010) Dietary glucosinolate intake, polymorphisms in selected biotransformation enzymes, and risk of prostate cancer. Cancer Epidemiol Biomarkers Prev 19: 135–143. 20056632 10.1158/1055-9965.EPI-09-0660

[68] Rodrigues IS, Kuasne H, Losi-Guembarovski R, Fuganti PE, Gregório EP, et al (2011) Evaluation of the influence of polymorphic variants CYP1A1 2B, CYP1B1 2, CYP3A4 1B, GSTM1 0, and GSTT1 0 in prostate cancer. Urol Oncol 29: 654–663. 20884258 10.1016/j.urolonc.2010.01.009

[69] Lavender NA, Benford ML, VanCleave TT, Brock GN, Kittles RA, et al (2009) Examination of polymorphic glutathione S-transferase (GST) genes, tobacco smoking and prostate cancer risk among men of African descent: a case-control study. BMC Cancer 9: 397. 19917083 10.1186/1471-2407-9-397 PMC2783040

[70] Safarinejad MR, Shafiei N, Safarinejad SH (2011) Glutathione S-transferase gene polymorphisms (GSTM1, GSTT1, GSTP1) and prostate cancer: a case-control study in Tehran, Iran. Prostate Cancer Prostatic Dis 14: 105–113. 21243008 10.1038/pcan.2010.54

[71] Lima MM Jr, Oliveira MN, Granja F, Trindade AC, De Castro Santos LE, et al (2008) Lack of association of GSTT1, GSTM1, GSTO1, GSTP1 and CYP1A1 polymorphisms for susceptibility and outcome in Brazilian prostate cancer patients. Folia Biol (Praha) 54: 102–108. 18647550 10.14712/fb2008054030102

[72] Souiden Y, Mahdouani M, Chaieb K, Elkamel R, Mahdouani K (2010) Polymorphisms of glutathione-S-transferase M1 and T1 and prostate cancer risk in a Tunisian population. Cancer Epidemiol 34: 598–603. 20599479 10.1016/j.canep.2010.06.002

[73] Sivonová M, Waczulíková I, Dobrota D, Matáková T, Hatok J, et al (2009) Polymorphisms of glutathione-S-transferase M1, T1, P1 and the risk of prostate cancer: a case-control study. J Exp Clin Cancer Res 28: 32. 19265530 10.1186/1756-9966-28-32 PMC2654432

[74] Kwon DD, Lee JW, Han DY, Seo IY, Park SC, et al (2011) Relationship between the Glutathione-S-Transferase P1, M1, and T1 Genotypes and Prostate Cancer Risk in Korean Subjects. Korean J Urol 52: 247–252. 21556210 10.4111/kju.2011.52.4.247 PMC3085616

[75] Sahar Ansari B, Vasudevan R, Mirinargesi M, Patimah I, Sabariah AR, et al (2009) Lack of association of glutathione S-transferase gene polymorphisms in Iranian prostate cancer subjects. Am J Biochem & Biotech 5: 30–34.

[76] Wang YL, Jiang J, Wang LF, Liu YF (2005) Polymorphisms of glutathione-S-transferase genes GSTM1 and GSTT1 and prostate cancer risk in Chinese population. Acta Academiae medicinae militaris tertiae 10: 1039–1041.

[77] Xu XX, Chang WJ, Hou JG, Xu DF, Cui XG, et al (2010) Relationship of GSTP1,RASSF1A polymorphisms and environmental agent with susceptibility to prostate cancer: a case-control study. Academic Journal of Second Military Medical University 1: 12–17.

[78] Wang YL, Jiang J, Jin FS, Wang LF, Wan JH (2008) Polymorphisms of glutathione-S-transferase gene Pi (GSTP1) and prostate cancer risk in Chinese population. Shanxi Med J 5: 410–412.

[79] Hemelrijck MV, Rohrmann S, Steinbrecher A, Kaaks R, Teucher B, et al (2012) Heterocyclic Aromatic Amine [HCA] Intake and Prostate Cancer Risk: Effect Modification by Genetic Variants. Nutr Cancer 64: 704–713. 22564066 10.1080/01635581.2012.678548

[80] Catsburg C, Joshi AD, Corral R, Lewinger JP, Koo J, et al (2012) Polymorphisms in carcinogen metabolism enzymes, fish intake, and risk of prostate cancer. Carcinogenesis 33: 1352–1359. 22610071 10.1093/carcin/bgs175 PMC3499053

[81] Qadri Q, Sameer AS, Shah ZA, Hamid A, Alam S, et al (2011) Genetic polymorphism of the glutathione-S-transferase P1 gene (GSTP1) and susceptibility to prostate cancer in the Kashmiri population. Genet Mol Res 10: 3038–3045. 22180037 10.4238/2011.December.6.4

[82] Foley R, Hollywood D, Lawler M (2004) Molecular pathology of prostate cancer: the key to identifying new biomarkers of disease. Endocr Relat Cancer 11: 477–488. 15369449 10.1677/erc.1.00699

[83] Strange RC, Spiteri MA, Ramachandran S, Fryer AA (2001) Glutathione-S-transferase family of enzymes. Mutat Res 482: 21–26. 11535245 10.1016/s0027-5107(01)00206-8

[84] Xue H, Su J, Sun K, Xie W, Wang H (2011) Glutathione S-transferase M1 and T1 gene polymorphism and COPD risk in smokers: an updated analysis. Mol Biol Rep 39: 5033–5042. 22160572 10.1007/s11033-011-1300-6

[85] Marcos M, Pastor I, Chamorro AJ, Ciria-Abad S, González-Sarmiento R, et al (2011) Meta-analysis: glutathione-S-transferase allelic variants are associated with alcoholic liver disease. Aliment Pharmacol Ther 34: 1159–1172. 21967547 10.1111/j.1365-2036.2011.04862.x

[86] Wang J, Zou L, Huang S, Lu F, Lang X, et al (2010) Genetic polymorphisms of glutathione S-transferase genes GSTM1, GSTT1 and risk of coronary heart disease. Mutagenesis 25: 365–369. 20354063 10.1093/mutage/geq014

[87] Choubey VK, Sankhwar SN, Tewari R, Sankhwar P, Singh BP, et al (2012) Null genotypes at the GSTM1 and GSTT1 genes and the risk of benign prostatic hyperplasia: A case-control study and a meta-analysis. Prostate: 1–7. Available: http://dx.doi.org/10.1002/pros.22549. 10.1002/pros.22549 22692893

[88] Zintzaras E, Ioannidis JP (2005) Heterogeneity testing in meta-analysis of genome searches. Genet Epidemiol 28: 123–137. 15593093 10.1002/gepi.20048

[89] Lin PI, Vance JM, Pericak-Vance MA, Martin ER (2007) No gene is an island: the flip-flop phenomenon. Am J Hum Genet 80: 531–538. 17273975 10.1086/512133 PMC1821115

